# Three New Truffle Species (*Tuber*, *Tuberaceae*, *Pezizales*, and *Ascomycota*) from Yunnan, China, and Multigen Phylogenetic Arrangement within the Melanosporum Group

**DOI:** 10.3390/jof10090640

**Published:** 2024-09-07

**Authors:** Rui Wang, Gangqiang Dong, Yupin Li, Ruixue Wang, Shimei Yang, Jing Yuan, Xuedan Xie, Xiaofei Shi, Juanbing Yu, Jesús Pérez-Moreno, Fuqiang Yu, Shanping Wan

**Affiliations:** 1 College of Resources and Environment, Yunnan Agricultural University, Kunming 650100, China; nyyy730@163.com (R.W.); liyupin2021@163.com (Y.L.); wlshizuibang@163.com (R.W.); yangshimei@mail.kib.ac.cn (S.Y.); yuanjing@mail.kib.ac.cn (J.Y.); y0520152015@163.com (J.Y.); 2 Amway (China) Botanical R&D Center, Wuxi 214115, China; tony.dong@amway.com; 3 The Germplasm Bank of Wild Species, Yunnan Key Laboratory for Fungal Diversity and Green Development, Kunming Institute of Botany, Chinese Academy of Sciences, 132 Lanhei Road, Kunming 650201, China; shixiaofei@mail.kib.ac.cn; 4 Herbarium, Key Laboratory for Plant Diversity and Biogeography of East Asia, Kunming Institute of Botany, Chinese Academy of Sciences, Kunming 650201, China; xiexuedan@mail.kib.ac.cn; 5 Colegio de Postgraduados, Campus Montecillo, Microbiología, Edafología, Texcoco 56230, Mexico; jepemo@yahoo.com.mx

**Keywords:** black truffles, phylogeny, species definition, taxonomy

## Abstract

Based on a multi-locus phylogeny of a combined dataset of ITS, LSU, *tef1-*α, and *rpb2* and comprehensive morphological analyses, we describe three new species from the Melanosporum group of genus *Tuber* and synonymize *T. pseudobrumale* and *T. melanoexcavatum*. Phylogenetically, the three newly described species, *T. yunnanense*, *T. melanoumbilicatum* and *T. microexcavatum*, differ significantly in genetic distance from any previously known species. Morphologically, *T. yunnanense* is distinctly different from its closest phylogenetically related species, *T. longispinosum*, due to its long shuttle-shape spores (average the ratio of spore length to spore width for all spores (Qm) = 1.74). *Tuber melanoumbilicatum* differs from the other species in having a cavity and long shuttle-shaped spores (Qm = 1.65). Although *T. microexcavatum* sampled ascomata have relatively low maturity, they can be distinguished from its closely related species *T. pseudobrumale* by the ascomata size, surface warts, and spore number per asci; additionally, phylogenetic analysis supports it as a new species. In addition, molecular analysis from 22 newly collected specimens and Genebank data indicate that *T. pseudobrumale* and *T. melanoexcavatum* are clustered into a single well-supported clade (Bootstrap (BS) = 100, posterior probabilities (PP) = 1.0); and morphological characteristics do not differ. Therefore, based on the above evidence and publication dates, we conclude that *T. melanoexcavatum* is a synonym of *T. pseudobrumale*. By taking into account current knowledge and combining the molecular, multigene phylogenetic clade arrangement and morphological data, we propose that the Melanosporum group should be divided into four subgroups. Diagnostic morphological features and an identification key of all known species in the Melanosporum group are also included. Finally, we also provide some additions to the knowledge of the characterization of *T. pseudobrumale*, *T. variabilisporum,* and *T. pseudohimalayense* included in subgroup 1 of the Melanosporum group.

## 1. Introduction

*Tuber* F.H. Wigg. (*Tuberaceae*, *Pezizales*) is renowned for its distinctive aroma, flavor, and nutritional benefits, including high fiber, protein, and low fat [1,2,3]. Some species of this genus are among the most highly prized edible wild mushrooms in international markets including mainly *T*. *magnatum* Pico and *T. melanosporum* Vittad. [4]. The genus *Tuber* is species-rich and geographically widespread, with over 180 species in the world mainly distributed in Europe, North America, and Southeast Asia [5]. In China, 82 phylogenetic species have been recorded, 68 of which are only found in this country, showing a high rate of endemism [6]. *Tuber* establish typical ectomycorrhizal symbioses with woody plant species belonging mainly to *Pinaceae*, *Fagaceae*, *Corylaceae*, and *Betulaceae* [7,8,9]. This relationship plays a role of paramount importance in the functioning of forest systems because it enhances plant nutrient absorption and resilience and also has a crucial role in plant–fungus–animal coevolution [10,11,12,13].

The study of the genus *Tuber* originated in Europe and the United States, where the Melanosporum group is considered one of the most important genealogical branches of the genus because of its food value, species diversity, and wide geographical distribution [5,14,15,16,17,18]. In terms of the timeline of species descriptions, the earliest described species of black truffle is *T. melanosporum*, which was described by the French mycologist Jean Louis Étienne Alexandre Brongniart in 1813 [19]. Since then, *T. melanosporum* has been one of the most studied species within the *Tuber* genus, attracting significant attention from researchers and food enthusiasts alike due to its culinary and economic value and its wide geographical distribution [2,20,21]. In 1831, the Italian botanist Carlo Vittadini provided a systematic description of species within the *Tuber* genus in his work “Monographia Tuberacearum”, which included *T. brumale* [22]. As one of the most widely distributed black truffles globally, *T. brumale* plays a crucial role in taxonomic studies of *Tuber* through morphological and molecular analyses [20,23]. In the late 19th century, the earliest Asian black truffle species was discovered in India. In 1892, Duthie collected a species resembling the *T. melanosporum* under *Quercus incana* Roxb. in the Mussoorie region of northern India. These specimens were sent to the Royal Botanic Gardens, Kew, and subsequently published as a new species, *T. indicum* Cooke and Massee [24]. In the 1980s, Zhang studied Chinese specimens resembling the type specimen of *T. indicum*. Based on the ornamentation of the spores, individuals primarily characterized by spiny ornamentation were identified as *T. indicum*, while those with regular or irregular reticulate ornamentation on the spores were described as a new species, named *T. himalayense* Zhang and Minter [25]. In 1988, Tao collected black truffles in Sichuan, China, and the following year, published *T. sinense* K. Tao and B. Liu [26]. Concurrently, Hu discovered a black truffle species under *Q. glauca* Thunb. in Nantou County, Taiwan, and named it *T. formosanum* H. T. Hu [27]. However, the publication did not specify a type specimen, unfortunately rendering it an invalid species name. It was not until 2013 that the species was formally published as *T. formosanum* by H. T. Hu and Y. Wang [28]. The categorization and naming of Asian black truffles, particularly those originating from China, have historically been subject to confusion and debate. In 1999, Roux et al. analyzed Asian black truffles from China using ITS fragments and found that these samples were divided into two phylogenetic distinct branches, representing *T. indicum* and *T. himalayense* [29]. Later scholars, including Zhang et al. and Feng et al. in China and Kinoshita et al. in Japan, held the same view on the interpretation of these two branches [30,31,32]. Meanwhile, *T. sinense* and *T. formosanum* are considered to be homonyms of *T. indicum* and *T. himalayense*, respectively. There are also scholars who have not agreed with the interpretation of these two branches and have replaced them as *T. indicum*-A and *T. indicum*-B [15,16,33]. In this classification’s proposal, *T. sinense* and *T. himalayense* are treated as synonyms of *T. indicum* [34]. One of the reasons for the confusion in the classification of black truffle species is that their morphological characteristics are highly variable, and a single morphological method cannot identify species with a high degree of similarity [30,32,34,35]. By 2018, phylogenetic analyses of more than 200 rDNA-ITS sequences from different parts of Asia by Fan et al. showed that the black truffle species cluster in Asia contained at least four species, with three branches containing the type sequences of *T. sinense*, *T. formosanum,* and *T. yigongense* L. Fan and W. P. Xiong, from China, respectively, and one branch containing *T. longispinosum* A. Kinosh. from Japan [32,35]. According to recent studies, the Asian black truffle species group is reported to consist of six species, of which four, namely *T. formosanum*, *T. sinense*, *T. yigongense,* and *T. longispinosum*, are supported by molecular evidence, while no plausible DNA data were available for *T. indicum* and *T. himalayense* samples of Indian origin [35]. In addition to the closely related species mentioned above, there are also species from one branch of the Melanosporum group that are vaguely delimited. Moreno et al. described *T. pseudohimalayense* G. Moreno, Manjón, Diez, and Garcia-Montero [36] and Wang et al. [37] described *T. pseudoexcavatum* Y. Wang and G. Moreno, but it has been concluded that *T. pseudohimalayense* and *T. pseudoexcavatum* are a single species [38,39]. While Asian black truffle species were discovered, Guevara et al. identified *T. regimontanum* Guevara, Bonito and Rodríguez in Mexico [40]. In recent years, three black truffle species, *T. pseudobrumale* Y. Wang and S. H. Li, *T. melanoexcavatum* Y. Wang and S. H. Li, and *T. variabilisporum* L. Fan and T. Li were described from southwestern China [6,41,42]. Therefore, currently, the Melanosporum group contains 11 species supported by molecular data.

In our recent investigations, we collected a number of black truffle species and conducted a combined detailed analysis of their morphology and polygenic sequences and compared them with those of previously known species. Our aim was to define the species diversity and phylogenetic relationships within the Melanosporum group. The results revealed the discovery of three new species, namely *T. yunnanense* S. P. Wan, R. Wang and F.Q. Yu, sp. nov. and *T. melanoumbilicatum* S. P. Wan, R. Wang, and F.Q. Yu, sp. nov., which were morphologically mature, and *T. microexcavatum* S. P. Wan, R. Wang, and F.Q. Yu, sp. nov., which lacks mature spores and is described solely by its macro- and micromorphological characterization of ascomata, peridium, and gleba as well as the immature spores and asci, along with the sequences of four genes sequenced from its DNA. Interestingly, our findings also indicated that the morphological phylogenetic analyses indicate that *T. melanoexcavatum* is a synonym of *T. pseudobrumale*.

## 2. Materials and Methods

### 2.1. Morphological Study

Fresh samples were collected from Yunnan, China. The macromorphological description was based on fresh ascomata and microscopic and macroscopic characteristics were described following the methods of Kumar et al. [43]. Hand-cut sections were mounted in 5% (w/v) KOH and examined under a light microscope (Leica DM2500, Leica Microsystems, Wetzlar, Germany). To assess the range of spore size, 376 ascospores were measured from *T. yunnanense* specimens and 423 from *T. melanoumbilicatum* specimens. Measurements of ascospores are given as (a–) b–c(–d), where b–c includes a minimum of 90% of the measured values. Extreme values (a and d) are given in parentheses. The abbreviation “Q” represents the ratio range of spore length to spore width calculated for each spore and “Qm ± ssd (sample standard deviation)” for the average Q of all spores ± ssd. For scanning electron microscopy (SEM), spores were scraped from the dried gleba onto double-sided tape and this was mounted directly on an SEM stub, coated with gold-palladium, and examined and photographed using a JSM-5600LV SEM (JEOL, Tokyo, Japan). The specimens are deposited at the Herbarium of Yunnan Agricultural University (YNAU).

In the present study, we conducted a comparative analysis of the morphological characters of all species of the Melanosporum group. Additionally, we studied the morphological features of 22 collected specimens resembling *T. pseudobrumale* in detail (Table 1). The ascomata surface ornamentation, peridium, asci, and ascospores of the 22 specimens were studied in detail, and at least 98 spores were measured for each specimen. A detailed morphological description of the harvested specimens was provided to further elucidate the morphology of *T. pseudobrumale*. Due to their recorded natural morphological variation, we also include photographs of the ascomata of two additional species: *T. variabilisporum* and *T. pseudohimalayense*.

### 2.2. Molecular Methods

The total DNA was extracted from pieces of dried ascomata with a modified CTAB procedure [44]. Standard and touchdown polymerase chain reaction (PCR) protocols along with fungal-specific primer sets were used to amplify and sequence four regions: the internal transcribed spacer of ribosomal RNA gene (ITS), the 28S large subunit of ribosomal RNA gene (LSU), elongation factor (*tef1-*α), and the second largest subunit of RNA polymerase II (*rpb2*) [5].

The PCR was performed using the following procedure: 25 μL of PCR reaction solution contained 1 μL DNA (50 ng/μL), 1 μL (5 μm) of each primer pair, 2.5 μL 10 × buffer (Mg^2+^plus), 1 μL dNTP (1 mM), 0.5 μL BSA (0.1%), 0.5 μL MgCl_2_ (2.0 mM), and 1 U of Taq DNA polymerase (Takara Tag, Takara Biotechnology, Dalian, China). The thermal cycling conditions were run as follows: an initial denaturation at 94 °C for 5 min, followed by 35 cycles of 94 °C for 1 min, annealing at 52 °C for 1 min, and 72 °C for 1 min. The final reaction was followed by extension at 72 °C for 10 min. The PCR products were verified on 1% agarose electrophoresis gels stained with ethidium bromide. The PCR products were subsequently purified and sequenced by Tsingke Biotech Corporation (Beijing, China). The obtained alignment and respective phylogenetic tree were deposited in the TreeBASE with the submission ID 31641.

### 2.3. Sequence Analysis

Sequences (ITS, LSU, *tef1-*α, and *rpb2*) from the studied specimens were compiled along with sequences from reference taxa curated in GenBank (http://www.ncbi.nlm.nih.gov/ (accessed on 12 October 2023)). A total of 109 taxa including holotypes were analyzed (Table A1). A dataset (ITS, LSU, *tef1-*α, and *rpb2*) was used to clarify the phylogenetic position of the new species. Two sequences derived from *Choiromyces sichuanense* S. P. Wan, R. Wang, and F.Q. Yu were selected and used as an outgroup.

Datasets were aligned using Multiple Alignment using Fast Fourier Transform (MAFFT) v.7.0 [45] and then manually edited with BioEdit v.7.0.9 as needed [46]. Phylogenetic relationships among the taxa were inferred using Maximum likelihood (ML) and Bayesian inference (BI) methods. ML bootstrap (BS) replicates (1000) were computed in Randomized Axelerated Maximum Likelihood (RAxML) with a rapid bootstrap analysis and a search for the best-scoring ML tree. Bayesian analysis was conducted using the selected model, with four chains sampled every 100 generations, over a total of 3,000,000 generations. The average standard deviations of split frequencies were less than 0.01 at the end of the run and effective sampling size (ESS) values exceeded 200. A majority rule consensus tree was built after discarding trees from 25% of the initial trees as burn-in. Posterior probabilities (PP) were calculated using the sumt command implemented in MrBayes.

**Table 1:** Morphological characteristics of all species within the Melanosporum group.

Cavity	Fungal Taxa	Voucher Specimens	Ascomata Surface		Asci Spore Number	Ascospores			Source
			Color	Warts		Q Interval of All Spores, Average Value of All Spores and Main Shape	Size (μm)	Ornamentation	
Yes	*Tuber brumale*	—	black	penta- or hexagonal flat warts	3–5	nd Qm = 1.6 ± 0.1, mainly long ellipsoid	(25.3)–28.1–(33.7) × (15.7)–17.4–(19.1) μm	3.4 ± 0.38 μm, spino-reticulate	Vittadini (1831) [22] Wang et al. (2006) [34]
	*Tuber pseudohimalayense*	AH 18331	black	Pyramidal	1–7(8)	nd Qm = 1.30 ± 0.09, mainly broadly ellipsoidal,	1-spored: 34–35 × 25–30 μm 2-spored: 30–34 × 22–30 μm 3–7-spored: — (including the ornamentation)	4–6(–8) μm, spino-reticulate	Moreno et al. (1997) [36] Wang et al. (2006) [34] Chen & Liu (2011) [33]
	*Tuber melanoumbilicatum*	YNAU017 holotype	black	solid, irregular polygonal, pyramidal warts	(1–)2–7	Q = 1.2–2.0, Qm = 1.65 ± 0.1, mainly long ellipsoid	1-spored: (42.5–)44.6–48.8(–54.5) × (25.7–)26.6–29.1(–30.2) μm 2-spored: (26.3–)30.9–41.4(–42.2) × (18.2–)18.5–23.9(–24.6) μm 3-spored: (19.2–)20.9–35.4(-36.9) × (13.7–)15.4–22.2(–26.2) μm 4-spored: (21.5–)24.3–31.7(–32.2) – (14.2–)14.9–20.2(–21.4) μm 5-spored: (21.7–)22.2–29.9(–33.1) × (13.1–)14.2–18.1(–20.2) μm 6-spored: (19.2–)21.7–28.0(–29.4) × (11.7)13.1–17.5(–18.9) μm 7-spored: (16.8–)18.9–26.8(–27.4) × (8.8–)12.5–16.3(–16.8) μm	0.9–8.2 μm, spino-reticulate	This study
	*Tuber pseudobrumale*	YAAS L3181 holotype	black	low pyramidal warts	3–7	nd Q = 1.27, mainly ellipsoid	3-spored: 26–30 × 15.5–17.5 μm 4-spored: (22)23–25.5(27) × 14–17 μm 5-spored: (21)22–25(25.4) × 13.5–15(16) μm 6-spored: 21–23.5 × 13–14.5 μm 7-spored: 21–22.5 × 12–14 μm	4–5 μm spino-reticulate	Li et al. (2014) [41]
	*Tuber melanoexcavatum*	YAAS L3605 holotype	black	pyramidal warts	5–8	nd Q = 1.19, mainly ellipsoid,	5-spored: 22–24.7 × 15.4–16.9 μm 6-spored: 21.4–24 × 14.3–16.0 μm 7-spored: 20–22 × 13.5–15.5 μm 8-spored: 18.7–21.2 × 12.6–15.1 μm	3–4 μm spino-reticulate	Wang et al. (2020) [42]
	*Tuber pseudobrumale*	(22 samples shown on Table A1)	black	solid, low, concave, irregular, polygonal warts	1–6(–8)	Q = 1.1–1.9, Qm = 1.45 ± 0.2, malily ellipsoid	1-spored: (19.1–)22.4–42.2(–52.3) × (13.8–)16.2–27.8(–36.7) μm 2-spored: (17.5–)21.8–36.3(–42.5) × (12.6–)16.1–23.9(–31.9) μm 3-spored: (13.8–)20.1–26.1(–40.6) × (12.4–)14.7–21.8(–29.0) μm 4-spored: (13.3–)17.9–28.3(–33.9) × (9.6–)13.5–19.3(–26.1) μm 5-spored: (13.0–)17.3–26.5(–32.0) × (9.6–)12.6–18.4(–22.8) μm 6-spored: (11.5–)16.7–25.4(–33.5) × (9.1–)12.3–18.2(–23.3) μm 7-spored: (9.6–)12.8–23.7(–28.4) × (5.6–)9.6–16.1(–18.1) μm 8-spored: (15.9–)18.2–21.9(–22.7) × (10.5–)12.4–15.1(–15.7) μm	0.4–9.6 μm, spino-reticulate	This study
	*Tuber variabilisporum*	BJTC FAN362 holotype	dark brown to black brown	verrucose	1–5(–6)	Q = 1.06–1.44, nd broadly ellipsoid and ellipsoid	1-spored: 30–37.5 × 21.75–27.5 μm 2-spored: 27.5–32.5 ×20–23.5 μm 3-spored: 22.5–32.5 × 18.75–21.25 μm 4-spored: 17.5–25.5 ×16.5–20 μm 5-spored: 16.5–22.5 × 13.25–17.5 μm	3–5 μm, spino-reticulate	Fan et al. (2022) [6]
	*Tuber microexcavatum*	YNAU 1263 holotype	yellowish brown	loose-textured, cracked irregular warts	1–6	nd nd ellipsoid	nd	nd	This study
	*Tuber* sp. 5	K229	black	pyramidal warts	5–8	Q = 1.0–2.0, nd ellipsoid	15–20 × 10–15 μm	nd spino-reticulate	Kinoshita et al. (2011) [17]
No	*Tuber longispinosum*	K447 holotype	brown to dark greyish	low polygonal warts	1–5(–6)	Q = 1.0–2.1, nd ellipsoid to subglobose	1-spored: 31–41 × 22–30 μm 2-spored: 21–38 × 16–29 μm 3-spored: 19–34 × 15–26 μm 4-spored: 15–33 × 13–22 μm 5-spored: 16–31 × 12–20 μm 6-spored: 15–26 × 13–18 μm	3–7 (–12) μm, spiny	Kinoshita et al. (2018) [32]
	*Tuber yunnanense*	YNAU019 holotype	dark brown to black	solid, irregular polygonal, clustered pyramidal ridged warts	1–5(–6)	Q = 1.1–2.2, Qm = 1.74 ± 0.1, malily long shuttle-shaped	1-spored: (31.8–)32.3–52.8(–54.6) × (19.1–)20.2–33.1(–35.4) μm 2-spored: (25.4–)30.9–39.8(–43.6) × (15.7–)17.1–22.7(–25.1) μm 3-spored: (26.7–)28.1–35.9(–43.3) × (14.7–)15.4–21.4(–29.4) μm 4-spored: (25.1–)26.1–31.6(–32.7) × (14.3–)14.7–18.3(–20.6) μm 5-spored: (20.0–)20.6–28.2(–28.6) × (11.6–)12.8–17.1(–18.3) μm 6-spored: (22.5–)23.1–28.8(–29.4) × (11.7–)14.0–18.6(–19.2) μm	0.7–11.1 μm, spiny	This study
	*Tuber regimontanum*	ITCV 909 holotype	dark brown to black	pyramidal verrucae	1–4	nd nd broadly fusiform to ellipsoid.	1-spore: 40–55 (–62) × 30–31 μm 2-spore: 37–42 × 25–26 μm 3-spore: 33–37 × 23–26 μm 4-spore: 28–35 × 18–22 μm	2–5 × 1–2 µm, spino-reticulate	Guevara et al. (2008) [40]
	*Tuber yigongense*	BJTC FAN731 holotype	dark brown to blackish	pentagonal and pyramidal warts	1–5	nd nd malily *ellipsoid*	1-spored: 35–45 × 25–30 μm 2-spored: 30–37.5 × 20–25 μm 3–5-spored: 20–32.5 × 17.5–22.5 μm	2.5–4 µm, densely spino-reticulate	Fan et al. (2018) [35]
	*Tuber sinense=* *T. indicum?*	MHSU 1633	brown, reddish brown or deeply brown	verrucose	1–4	nd nd malily ellipsoid	1-spore: 32–36.5 × 43–49.5 μm 2-spores: 26.5–30 × 39–45.5 μm 3-4-spores: 22.5–25 × 30–35 μm (including spines)	3–6(–7) µm, spiny	Tao et al. (1989) [26] Wang et al. (2006) [34] Chen (2007) [47] Fan et al. (2022) [6]
	*Tuber formosanum*	HKAS 62628	dark reddish brown to dark grayish brown	low pyramidal warts	1–4(–5)	Q = (1.17–)1.27–1.62(–1.70) nd malily ellipsoid	1-spored: (27–)29–45(–48) × 20–32(–35) μm 2-spored: (26–)27–36(–39) × (18–)19–24(–28) μm 3-spored: 24–34 × (16–)18–23(–25) μm 4-spored:(25–)26–32(–33) × (17–)18–22 μm	2–5(–6) µm, spino-reticulate	Qiao et al. (2013) [28]
	*Tuber melanosporum*	—	blackish	verrucose	1–5	Q = 1.4–2.1 nd malily ellipsoid	28–32 × 16–21 μm	2–4 μm.spiny, spino-reticulate	Vittadini (1831) [22] Wang et al. (2006) [34]

nd = not determined.

## 3. Results

### 3.1. Phylogenetic Analysis

**Figure 1:**
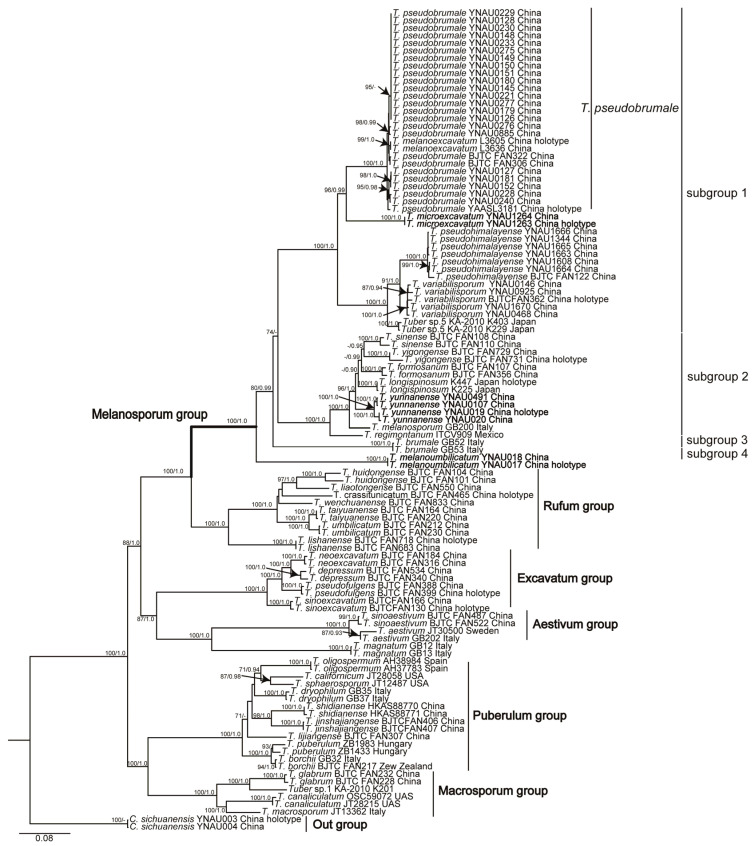
Randomized Axelerated Maximum Likelihood (RAxML) tree based on the sequences ITS, LSU, *tef1-*α, and *rpb2* of *T. yunnanense*, *T. melanoumbilicatum*, *T. microexcavatum,* and related species, with *C. sichuanensis* as the outgroup. Bootstrap (BS) values obtained from maximum likelihood (ML) analysis (≥70%) and posterior probabilities (PP) from Bayesian inference (≥0.90) are indicated above or below the branches at the nodes. Newly obtained sequences are highlighted in bold font.

Phylogenetic relationships were assessed using concatenated sequence data from four loci (ITS, LSU, *tef1-*α, and *rpb2*), totaling 3462 characters and including 109 representative sequences from various *Tuber* species. The Bayesian analysis yielded similar trees to the parsimony analysis; therefore, only the tree inferred from the ML analysis is shown in Figure 1.

Based on the sequences (ITS, LSU, *tef1-*α, and *rpb2*), all samples formed seven well-supported groups, representing the Melanosporum group, Rufum group, Excavatum group, Aestivum group, Puberulum group, Macrosporum group, and the outgroup (*C. sichuanensis*). All analyzed species in the Melanosporum group formed a monophyletic group with bootstrap support (BS = 100, PP = 1.0) and, based on the topology of the multigene phylogenetic analyses, we propose dividing them into four subgroups (termed then as subgroups 1, 2, 3, and 4).

The phylogenetic tree based on ITS, LSU, *tef1-*α, and *rpb2* datasets confirmed the presence of 14 phylogenetic species in the Melanosporum group (Figure 1). Each of the three new species (*T. microexcavatum*, *T. yunnanense,* and *T. melanoumbilicatum*) formed separate phylogenetic branches corresponding to subgroup 1, subgroup 2, and subgroup 4, respectively. *Tuber microexcavatum* was identified as a new species in subgroup 1 due to its 79.6% ITS similarity with the closely related *T. pseudobrumale*. ITS rDNA sequence analysis also showed that *T. pseudobrumale* and *T. melanoexcavatum* have similarities ranging from 98.0% to 99.7%, suggesting that they are the same species. Therefore, subgroup 1 comprised five species, with ITS similarities ranging from 70.5% to 94.5%. Another new species, *T. yunnanense*, formed a separate branch in subgroup 2 with strong support (BS = 100, PP = 1.0). Therefore, subgroup 2 included seven species, five of which are Asian species including *T. sinense*, *T. formosanum*, *T. yigongense,* and *T. yunnanense* from China and *T. longispinosum* from Japan. The other two species are the well-known European species *T. melanosporum* and the Mexican species *T. regimontanum*. The ITS similarities among the species in subgroup 2 ranged from 87.0% to 93.0%. Subgroup 3 included only the European species *T. brumale*, while subgroup 4 only included the new Chinese species *T. melanoumbilicatum*, ITS rDNA sequence analysis showed that the similarity between subgroups 3 and 4 was 70.7%.

### 3.2. Taxonomy

#### 3.2.1. *Tuber yunnanense* S. P. Wan, R. Wang and F.Q. Yu, sp. nov. Figure 2

MycoBank: MB849430

Etymology: Refers to the location of the type collection.

Typification: CHINA. Yunnan Province, Gongshan County, 29 October 2020, collected from *Pinus* sp., wsp973-1 (holotype YNAU019), dried specimens. GenBank: ITS = OK625306; LSU = OR661811; *tef1-*α = OR813081; *rpb2* = OR832407.

Diagnosis: Ascomata are black with solid irregular polygonal pyramidal warts on the surface. Pseudoparenchymatous peridium. Each ascus contains 1–5(–6) spores, and some asci have short stalks. Ascospores are predominantly long shuttle-ellipsoids that are golden yellow, measuring 20.0–54.6 × 17.1–35.4 μm, with sharp spines.

Description: Ascomata are dark brown to black, irregularly spherical, and range from 1.9–5.5 cm in diameter. The surface features grooves and is covered with black solid irregular polygonal pyramidal ridged warts, 0.4–1.3 mm high, with occasional cracks and depressions at the apex. The peridium is composed of two layers, pseudoparenchymatous; the outer layer is 103.5–193.6 μm thick, composed of irregular cells, 3.7–15.6 × 2.8–10.9 μm, yellowish-brown, or hyaline; the inner layer is 45.5–259.9 μm thick, composed of intricately interwoven hyaline and thin-walled hyphae that are 0.5–2.4 μm in diameter. The gleba is solid, brown to black when mature, and marbled with white veins. It is composed of hyaline and interwoven thin-walled hyphae, 0.8–1.5 μm broad at the septa; with cylindrical to inflated cells, 10.6–23.2 × 7.2–16.3 μm. Asci are irregularly shaped, 47.2–128.9 × 30.9–98.2 μm (*n* = 142), with size and shape varying depending on the number of ascospores, with 1–5(–6) spores per ascus. Most asci are sessile, with a few having short stalks, 4.8–8.2 × 5.2–7.7 μm (*n* = 4). Ascospores are golden yellow, ellipsoid, or subglobose. Spikes of different lengths are attached to the outer layer of each spore. Spore sizes are as follows: in 1-spored asci: (31.8–)32.3–52.8(–54.6) × (19.1–)20.2–33.1(–35.4) μm, Q = 1.4–2.0, Qm = 1.7 ± 0.15, spines = 1.2–11.1 μm (*n* = 40); in 2-spored asci: (25.4–)30.9–39.8(–43.6) × (15.7–)17.1–22.7(–25.1) μm, Q = 1.4–2.1, Qm = 1.8 ± 0.13, spines = 0.7–9.5 μm (*n* = 60); in 3-spored asci (26.7–)28.1–35.9(–43.3) × (14.7–)15.4–21.4(–29.4) μm, Q = 1.1–2.2, Qm = 1.8 ± 0.16, spines = 0.9–7.9 μm (*n* = 84); in 4-spored: (25.1–)26.1–31.6(–32.7) × (14.3–)14.7–18.3(–20.6) μm, Q = 1.5–2.1, Qm = 1.8 ± 0.12, spines = 0.7–7.1 μm (*n* = 120); in 5-spored asci: (20.0–)20.6–28.2(–28.6) × (11.6–)12.8–17.1(–18.3) μm, Q = 1.1–2.0, Qm = 1.7 ± 0.16, spines = 0.9–6.8 μm (*n* = 60); in 6-spored asci: (22.5–)23.1–28.8(–29.4) × (11.7–)14.0–18.6(–19.2) μm, Q = 1.5–1.9, Qm = 1.6 ± 0.12, spines = 0.8–4.9 μm (*n* = 12), spiny, 0.7–11.1 μm.

Additional material examined: CHINA, Yunnan Province, Gongshan County, 29 October 2020, collected from *Pinus* sp., wsp973-2 (YNAU020). GenBank: ITS = OK625307; LSU = OR661812; *tef1-*α = OR813082; *rpb2* = OR832408; ibid., wsp974-3 (YNAU0107). GenBank: ITS = OR665397; LSU = OR661813; *tef1-*α = OR813083; *rpb2* = OR832409; CHINA, Sichuan Province, 12 September 2021, collected from *Pinus* sp., wsp1365 (YNAU0491). GenBank: ITS = OR250186; LSU = OR661814; *tef1-*α = OR813084; *rpb2* = OR832410.

**Figure 2:**
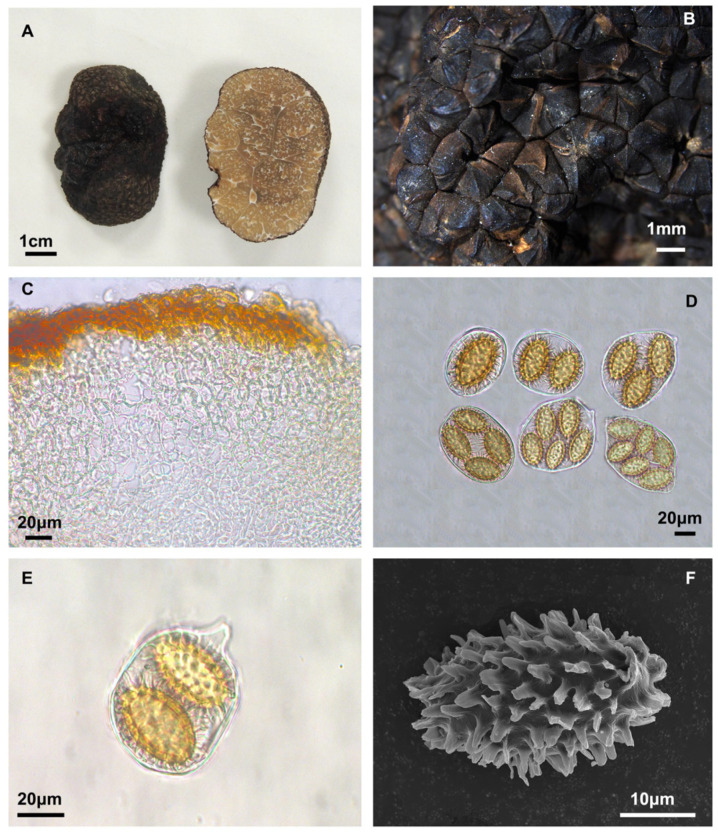
*Tuber yunnanense* (YNAU019, holotype). (**A**) Ascoma and gleba appearance; (**B**) Warts on surface ascoma; (**C**) Peridium hyphal arrangements; (**D**,**E**) Asci and ascospores under bright field microscopy; (**F**) Ascospore under scanning electronic microscopy. The scale bars are individually indicated for each image.

#### 3.2.2. *Tuber melanoumbilicatum* S. P. Wan, R. Wang, and F.Q. Yu, sp. nov. Figure 3

MycoBank: MB849431

Etymology: The Latin name *melanoumbilicatum* derives from ‘melano’ (black) referring to black ascomata and ‘umbilicatum’, indicating the navel-like structure of the ascomata.

Typification: CHINA. Yunnan Province, Baoshan City, 5 December 2020, collected from *Pinus* sp., wsp1006 (holotype YNAU017), dried specimens. GenBank: ITS = OK625304; LSU = OR661815; *tef1-*α = OR832379; *rpb2* = OR832411.

Diagnosis: Ascomata are black, with a distinct cavity, and are covered with black solid pyramidal warts. Pseudoparenchymatous peridium. Each ascus contains (1–)2–7 spores, sessile. Ascospores are predominantly long ellipsoid and black gold in color, measure 16.8–54.5 × 8.8–30.2 μm, and are spinoreticulate.

Description: Ascomata are black, with a distinct cavity, 2.7–3.5 cm in diameter. The surface is covered with black solid irregular polygonal sharp cones with verrucae convex 0.1–0.4 mm. The peridium is composed of two layers, pseudoparenchymatous; the outer layer, 142.3–255.4 μm thick, consists of subglobose to subangular cells, 6.6–29.5 × 3.5–11.5 μm, in yellow, pale brown, or hyaline; the inner layer, 55.1–133.4 μm thick, consists of intricately interwoven hyaline and thin-walled hyphae, 0.4–2.3 μm in diameter. The gleba is solid, white when young, and black when mature, marbled with white veins. It is composed of hyaline and interwoven thin-walled hyphae, 1.3–4.1 μm wide at the septa; with cylindrical to inflated cells, 3.8–10.8 × 3.5–6.7 μm. Asci are irregularly spherical and 61.5–81.9 × 47.4–72.9 μm (*n* = 106). Each ascus contains (1–)2–7 spores, sessile. Ascospores are black gold in color, mainly long ellipsoid, occasionally wide ellipsoidal, and long shuttle-shaped, with spines of varying lengths. Spore sizes are as follows: in 1-spored asci: (42.5–)44.6–48.8(–54.5) × (25.7–)26.6–29.1(–30.2) μm, Q = 1.5–1.9, Qm = 1.7 ± 0.15, spines = 2.2–8.0 μm (*n* = 5); in 2-spored asci: (26.3–)30.9–41.4(–42.2) × (18.2–)18.5–23.9(–24.6) μm, Q = 1.4–2.0, Qm = 1.7 ± 0.12, spines = 1.5–7.9 μm (*n* = 50); in 3-spored asci: (19.2–)20.9–35.4(–36.9) × (13.7–)15.4–22.2(–26.2) μm, Q = 1.2–2.0, Qm = 1.6 ± 0.16, spines = 1.2–8.2 μm (*n* = 48); in 4-spored asci: (21.5–)24.3–31.7(–32.2) × (14.2–)14.9–20.2(–21.4) μm, Q = 1.4–2.1, Qm = 1.7 ± 0.15, spines = 1.1–6.9 μm (*n* = 60); in 5-spored asci: (21.7–)22.2–29.9(–33.1) × (13.1–)14.2–18.1(–20.2) μm, Q = 1.2–2.0, Qm = 1.6 ± 0.15, spines = 0.9–6.5 μm (*n* = 100); in 6-spored asci: (19.2–)21.7–28.0(–29.4) × (11.7)13.1–17.5(–18.9) μm, Q = 1.3–2.0, Qm = 1.7 ± 0.13, spines = 0.9–6.3 μm (*n* = 90); and in 7-spored asci: (16.8–)18.9–26.8(–27.4) × (8.8–)12.5–16.3(–16.8) μm, Q = 1.2–2.0, Qm = 1.6 ± 0.15, spines = 0.9–6.0 μm (*n* = 70), spino-reticulate, reticulum with 6–10 meshes along the spore length and 6–8 across.

Additional material examined: CHINA, Yunnan Province, Baoshan City, 5 December 2020, collected from *Pinus* sp., wsp1006-1 (YNAU018). GenBank: ITS = OK625305; LSU = OR661816; *tef1-*α = OR832380; *rpb2* = OR832412.

**Figure 3:**
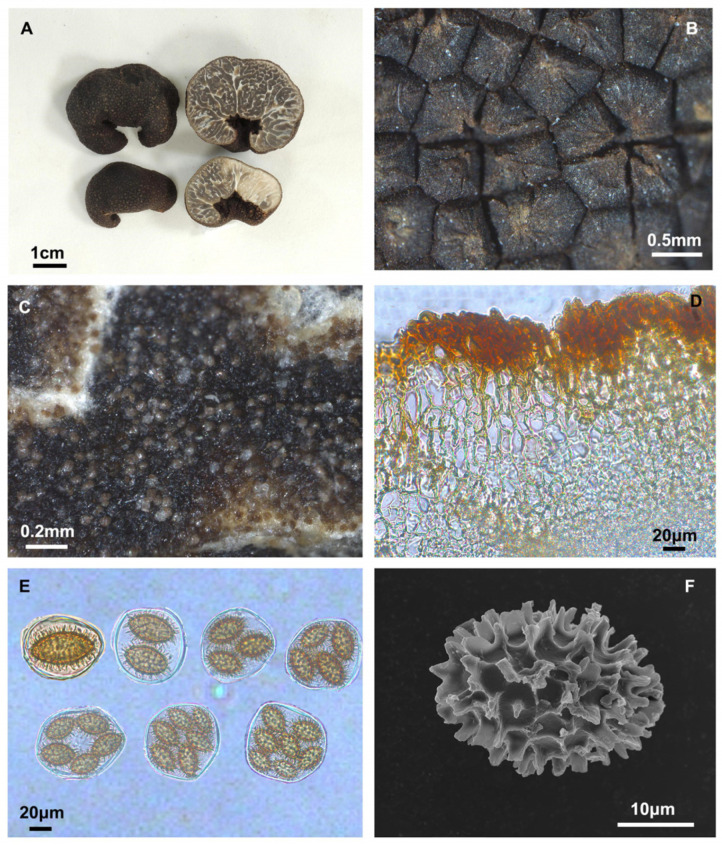
*Tuber melanoumbilicatum* (YNAU017, holotype). (**A**) Ascomatata and gleba in cross-section; (**B**) Warts on surface ascoma; (**C**) Close-up to gleba; (**D**) Peridium hyphal arrangement; (**E**) Asci and ascospores under bright field microscopy; (**F**) Ascospore under scanning electron microscopy. The scale bars are individually indicated for each image.

#### 3.2.3. *Tuber microexcavatum* S. P. Wan, R. Wang, and F.Q. Yu, sp. nov. Figure 4

MycoBank: MB854066

Etymology: The species name *microexcavatum* is derived from Latin ‘micro’ referring to the small ascomata and ‘excavatum’ referring to ascomata having a navel-like cavity.

Typification: CHINA. Yunnan Province, Luquan County, 11 August 2022, collected from *Platycarya strobilacea* Maxim., occasionally on *Pinus armandii* Franch., wsp2087 (holotype YNAU1263), dried specimens. GenBank: ITS = OR250184; LSU = OR661838; *tef1-*α = OR832381; *rpb2* = OR832413.

Diagnosis: Ascomata brown, with a distinct cavity, covered by yellow to brown loose-textured with cracked irregular warts. Pseudoparenchymatous peridium. Each ascus contains 1–6 spores, sessile.

Description: Ascomata has a distinct cavity, with a diameter of 0.7–0.9 cm. The surface is covered by yellow to brown loose-textured cracked irregular warts. The peridium is composed of two layers, pseudoparenchymatous; the outer layer that is 89.3–188.8 μm thick, composed of subglobose to subangular cells, 8.2–23.2 × 6.2–16.0 μm, yellowish brown, pale brown or hyaline; the inner layer is 137.6–224.7 μm thick, composed of intricately interwoven hyaline and thin-walled hyphae, 0.5–2.2 μm in diameter. The gleba is solid, marbled with white veins. It is composed of hyaline, interwoven, thin-walled hyphae, 1.1–3.4 μm broad at the septa, with cylindrical interwoven to inflated cells, 27.8–43.0 × 16.4–25.0 μm. Asci are irregularly spherical, 1–6 spored, sessile.

Additional material examined: CHINA, Yunnan Province, Luquan County, 11 August 2022, collected from *P. strobilacea*, occasionally on *P. armandii*, wsp2087-1 (YNAU1264). GenBank: ITS = OR250185; LSU = OR661839; *tef1-*α = OR832382; *rpb2* = OR832414.

**Figure 4:**
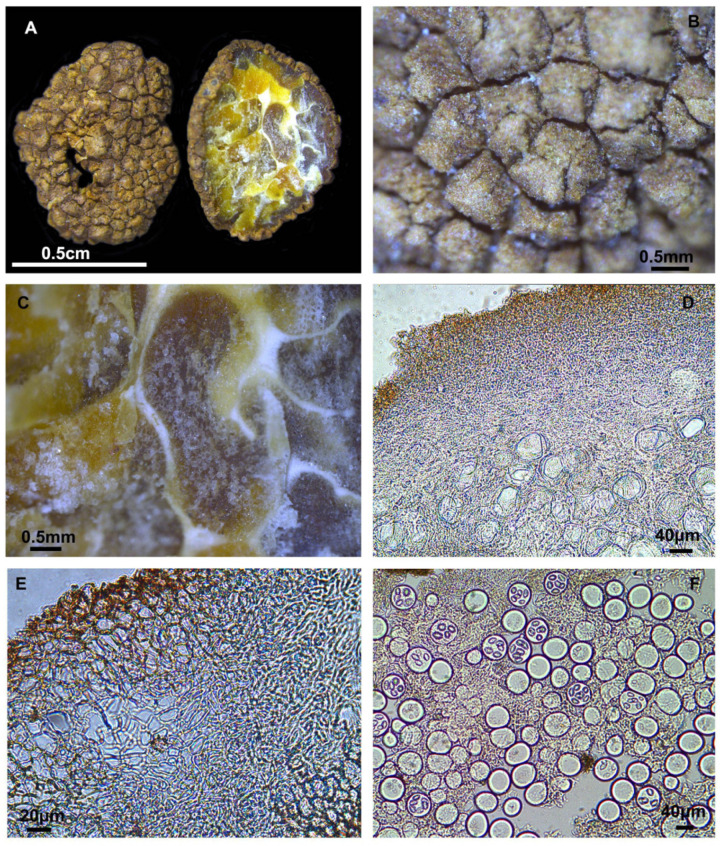
*Tuber microexcavatum* (YNAU1263, holotype). (**A**) Ascoma and gleba in cross-section; (**B**) Warts on surface ascoma; (**C**) Close-up to gleba; (**D,E**) Peridium hyphal arrangement; (**F**) Asci and ascospores under bright field microscopy. The scale bars are individually indicated for each image.

#### 3.2.4. *Tuber pseudobrumale* Y. Wang and Shu H. Li, *Mycol. Prog.* **2014**, *13*, 1157–1163 (Figure 5)

Description: Ascomata have a distinct cavity, measuring 1.3–2.0 cm in diameter. The surface is covered with sharp brownish-yellow cones. The peridium is composed of two layers, pseudoparenchymatous; the outer layer is 49.3–112.7 μm thick, composed of subglobose to subangular cells, 4.6–28.6 × 3.1–9.6 μm, that are yellow, pale brown or hyaline; the inner layer 24.9–71.6 μm thick, composed of intricately interwoven hyaline and thin-walled hyphae, 0.4–1.9 μm in diameter. The gleba is solid, yellowish brown when mature, and marbled with white veins. It is composed of hyaline interwoven thin-walled hyphae, 0.6–1.6 μm at the septa, with cells are cylindrical, interwoven, or inflated, 3.1–28.4 × 2.2–13.6 μm. Asci are irregularly spherical, 49.5–63.9 × 32.0–59.9 μm, with each containing 1–7(–8) spores (*n* = 115), sessile. Ascospores are brownish-yellow, mainly long ellipsoids, though occasionally they are subglobose or long shuttle-shaped and have spines of varying lengths. Spore sizes are as follows: in 1-spored asci: (29.5–)30.2–38.1(–38.8) × (17.3–)19.6–25.2(–26.1) μm, Q = 1.1–1.9, Qm = 1.6 ± 0.16, spines = 1.0–6.1 μm (*n* = 40); in 2-spored asci: (24.4–)25.9–31.9(–32.7) × (15.1–)15.7–20.4(–20.9) μm, Q = 1.4–2.0, Qm = 1.6 ± 0.13, spines = 1.2–5.9 μm (*n* = 40); in 3-spored asci: (20.0–)20.6–27.6(–29.9) × (12.4–)14.2–17.9(–19.0) μm, Q = 1.3–1.9, Qm = 1.6 ± 0.14, spines = 1.2–5.5 μm (n = 60); in 4-spored asci: (16.6–)19.1–26.1(–27.9) × (10.1–)13.5–17.0(–17.3) μm, Q = 1.1–2.0, Qm = 1.5 ± 0.14, spines = 1.2–5.5 μm (*n* = 60); in 5-spored asci: (16.3–)19.3–24.3(–26.4) × (11.6–)12.0–15.9(–16.3) μm, Q = 1.2–1.9, Qm = 1.5 ± 0.14, spines = 1.2–5.0 μm (*n* = 60); in 6-spored asci: (16.0–)17.3–22.8(–25.0) × (11.3–)11.9–15.1(–15.7) μm, Q = 1.2–1.7, Qm = 1.5 ± 0.12, spines = 1.2–5.0 μm (*n* = 72); in 7-spored asci: (15.1–)16.9–21.8(–23.3) × (9.6–)10.4–13.9(–16.0) μm, Q = 1.2–1.8, Qm = 1.5 ± 0.12, spines = 0.7–4.4 μm (*n* = 56); and in 8-spored asci: (15.9–)18.2–21.9(–22.7) × (10.5–)12.4–15.1(–15.7) μm, Q = 1.3–1.8, Qm = 1.5 ± 0.19, spines = 1.8–5.0 μm (*n* = 1), spino-reticulate, reticulum with 6–9 meshes along the spore length and 6–8 across.

Specimens examined: CHINA, Yunnan Province, 26 November 2020, collected from *Pinus* sp., wsp991 (YNAU0126). GenBank: ITS = OR665398; LSU = OR825717; *tef1-*α = OR832383; *rpb2* = OR832415; ibid., wsp992 (YNAU0127). GenBank: ITS = OR665399; LSU = OR661817; *tef1-*α = OR832384; *rpb2* = OR832416; ibid., wsp993 (YNAU0128). GenBank: ITS = OR665400; LSU = OR661818; *tef1-*α = OR832385; *rpb2* = OR832417; ibid., Weixi County, 5 December 2020, collected from *Pinus* sp., wsp1005 (YNAU0145). GenBank: ITS = OR665401; LSU = OR661819; *tef1-*α = OR832386; *rpb2* = OR832418; ibid., wsp1009 (YNAU0148). GenBank: ITS = OR665402; LSU = OR661820; *tef1-*α = OR832387; *rpb2* = OR832419; ibid., wsp1010 (YNAU0149). GenBank: ITS = OR665403; LSU = OR661821; *tef1-*α = OR832388; *rpb2* = OR832420; ibid., wsp1011 (YNAU0150). GenBank: ITS = OR665404; LSU = OR661822; *tef1-*α = OR832389; *rpb2* = OR832421; ibid., Baoshan City, 5 December 2020, collected from *Pinus* sp., wsp1012 (YNAU0151). GenBank: ITS = OR665405; LSU = OR661823; *tef1-*α = OR832390; *rpb2* = OR832422; ibid., wsp1013 (YNAU0152). GenBank: ITS = OR665406; LSU = OR661824; *tef1-*α = OR832391; *rpb2* = OR832423; ibid., Baoshan City, 20 December 2020, collected from *Pinus* sp., wsp1036 (YNAU0179). GenBank: ITS = OR665407; LSU = OR661825; *tef1-*α = OR832392; *rpb2* = OR832424; ibid., wsp1037 (YNAU0180). GenBank: ITS = OR665408; LSU = OR661826; *tef1-*α = OR832393; *rpb2* = OR832425; ibid., wsp1038 (YNAU0181). GenBank: ITS = OR665409; LSU = OR661827; *tef1-*α = OR832394; *rpb2* = OR832426; ibid., Xiangyun County, 20 December 2020, collected from *Pinus* sp., wsp1052 (YNAU0221). GenBank: ITS = OR665410; LSU = OR661828; *tef1-*α = OR832395; *rpb2* = OR832427; ibid., wsp1059 (YNAU0228). GenBank: ITS = OR665411; LSU = OR661829; *tef1-*α = OR832396; *rpb2* = OR832428; ibid., wsp1060 (YNAU0229). GenBank: ITS = OR665412; LSU = OR661830; *tef1-*α = OR832397; *rpb2* = OR832429; ibid., wsp1061 (YNAU0230). GenBank: ITS = OR665413; LSU = OR661831; *tef1-*α = OR832398; *rpb2* = OR832430; ibid., wsp1064 (YNAU0233). GenBank: ITS = OR665414; LSU = OR661832; *tef1-*α = OR832399; *rpb2* = OR832431; ibid., wsp1071 (YNAU0240). GenBank: ITS = OR665415; LSU = OR661833; *tef1-*α = OR832400; *rpb2* = OR832432; ibid., Weixi County, 2 January 2021, collected from *Pinus* sp., wsp1107 (YNAU0275). GenBank: ITS = OR665416; LSU = OR661834; *tef1-*α = OR832401; *rpb2* = OR832433; ibid., wsp1108 (YNAU0276). GenBank: ITS = OR665417; LSU = OR661835; *tef1-*α = OR832402; *rpb2* = OR832434; ibid., wsp1109 (YNAU0277). GenBank: ITS = OR665418; LSU = OR661836; *tef1-*α = OR832403; *rpb2* = OR832435; ibid., Weixi County, 31 October 2021, collected from *Pinus* sp., wsp1737 (YNAU0885). GenBank: ITS = OR665419; LSU = OR661837; *tef1-*α = OR832404; *rpb2* = OR832436.

**Figure 5:**
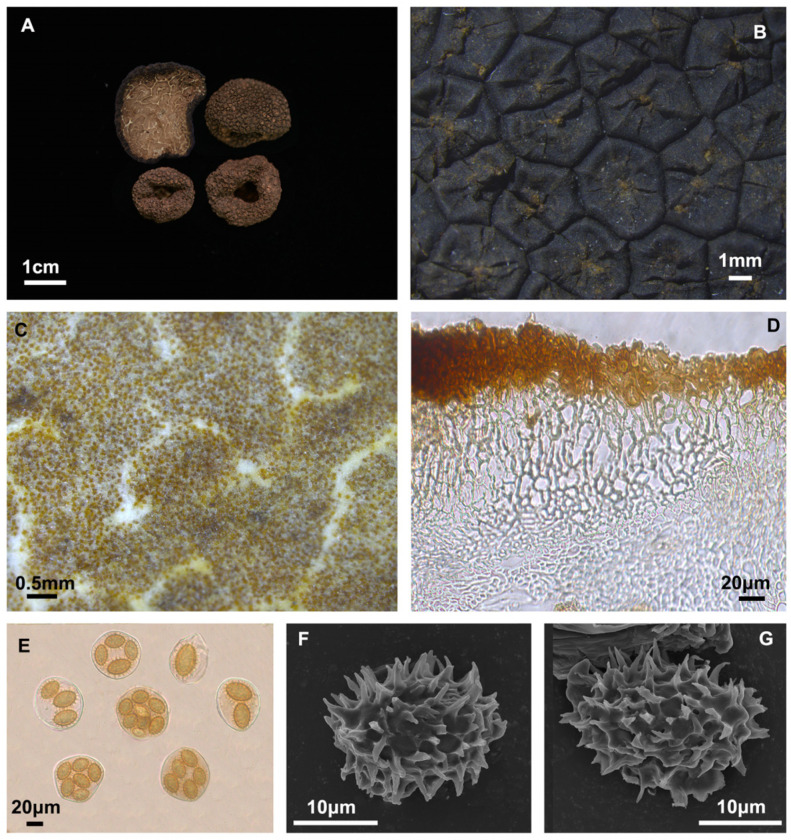
*Tuber pseudobrumale* (YNAU0221). (**A**) Ascomata and gleba in cross-section; (**B**) Warts on surface ascoma; (**C**) Close-up to gleba; (**D**) Peridium hyphal arrangement; (**E**) Asci and ascospores under bright field microscopy; (**F**,**G**) Ascospores under scanning electron microscopy. The scale bars are individually indicated for each image.

#### 3.2.5. *Tuber variabilisporum* L. Fan and T. Li, *Persoonia-Molecular Phylogeny and Evolution of Fungi*. **2022**, 48(1): 175–202 (Figure 6)

**Figure 6:**
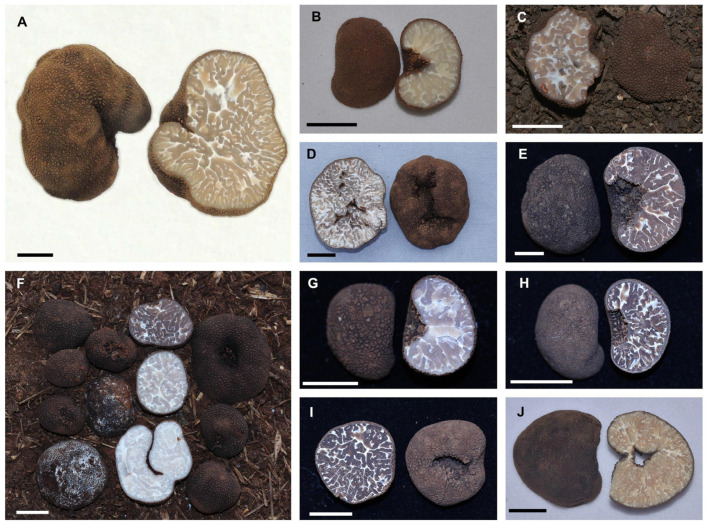
Ascomata of *T*. *variabilisporum* (**A**–**D**) and *T*. *pseudohimalayense* (**E**–**J**), showing cavities in their surfaces, with the exception of (**E**), which shows a less pronounced depression. (**A**) YNAU0146; (**B**) YNAU0468; (**C**) YNAU0925; (**D**) YNAU1670; (**E**) YNAU1664; (**F**) YNAU1608; (**G**) YNAU1663; (**H**) YNAU1665; (**I**) YNAU1666; (**J**) YNAU1344. (Scale bars = 1 cm).

Notes: In the original description, Fan et al. [6] pointed out that “*T. variabilisporum* is characterized by its … ascomata without basal cavity…”; however, morphological analyses from four sampled specimens identified molecularly as *T. variabilisporum* (Figure 6A–D), show noticeable depressions on three ascomata (Figure 6A,B,D) and the fourth specimen exhibited less pronounced depression (Figure 6C). Thus, it can be inferred that the surface of the *T. variabilisporum* is generally depressed but the possibility that some specimens show only a slight depression can occur. Molecular analyses confirm that our specimens and the holotype clustered into a single species branch with high support (BS = 100, PP = 1.0).

Specimens examined: CHINA, Yunnan Province, Dali City, 5 December 2020, collected from *Pinus* sp., wsp1007-1 (YNAU0146). GenBank: ITS = PP784759; LSU = PP784585; *tef1-*α = PP796847; *rpb2* = PP796857; ibid., Kunming City, 11 September 2021, collected from *Pinus* sp., wsp1346-1 (YNAU0468). GenBank: ITS = PP784760; LSU = PP784586; *tef1-*α = PP796848; *rpb2* = PP796858; ibid., Lijiang City, 24 November 2021, collected from *Q. glauca*, wsp1778 (YNAU0925). GenBank: ITS = PP784761; LSU = PP784587; *tef1-*α = PP796849; *rpb2* = PP796859; ibid., Chuxiong City, 12 December 2022, collected from *Pinus* sp., wsp2471 (YNAU1670). GenBank: ITS = PP784762; LSU = PP784588; *tef1-*α = PP796850; *rpb2* = PP796860.

#### 3.2.6. *Tuber pseudohimalayense* Moreno G, Díez M and García-Moreno, *Mycotaxon*. **1997**, 63: 217–224 (Figure 6)

Notes: Moreno et al. [36] initially described the presence of uncertain cavities on the surface of the ascomata of *T. pseudohimalayense*. Wang et al. [48] later described cavities on the ascomata of *T. pseudoexcavatum*. Later on, Chen et al. [39] confirmed that *T. pseudoexcavatum* is a synonym of *T. pseudohimalayense*. In the present study, we observed clear cavities on the ascomata of all six analyzed specimens of *T. pseudohimalayense* (Figure 6E–J), further confirming the presence of surface depressions. Moreover, specimens of *T. pseudohimalayense* collected in this study clustered with the type specimen into a single species with high support (BS = 100, PP = 1.0). Additionally, they were grouped with Chinese *T. pseudobrumale*, *T. microexcavatum*, *T. variabilisporum,* and Japanese *Tuber* sp. 5, all of which exhibit concave surfaces (Figure 1).

Specimens examined: CHINA, Yunnan Province, Kunming City, 8 September 2022, collected from *Pinus* sp., wsp2167 (YNAU1344). GenBank: ITS = PP784768; LSU = PP784594; *tef1-*α = PP796856; *rpb2* = PP796866; ibid., 22 November 2022, collected from *P. armandii*, wsp2411 (YNAU1608). GenBank: ITS = PP784763; LSU = PP784589; *tef1-*α = PP796851; *rpb2* = PP796861; ibid., Chuxiong City, 12 December 2022, collected from *Pinus* sp., wsp2465 (YNAU1663). GenBank: ITS = PP784764; LSU = PP784590; *tef1-*α = PP796852; *rpb2* = PP796862; ibid., wsp2466 (YNAU1664). GenBank: ITS = PP784765; LSU = PP784591; *tef1-*α = PP796853; *rpb2* = PP796863; ibid., wsp2467 (YNAU1665). GenBank: ITS = PP784766; LSU = PP784592; *tef1-*α = PP796854; *rpb2* = PP796864; ibid., wsp2467-1 (YNAU1666). GenBank: ITS = PP784767; LSU = PP784593; *tef1-*α = PP796855; *rpb2* = PP796865.

## 4. Discussion

The description and study of black truffle species began in the early 19th century. Over time, research has gradually deepened, encompassing various aspects such as morphology, geographical distribution, genetics, and ecology [6,21,22,49,50,51]. Brongniart first introduced the Melanosporum group, characterized by its black or brown appearance and irregular surface texture; some species have distinct basal cavities, releasing unique strong aromas, with two peridium layers. Asci containing multiple spores are usually large and elliptical, with spines of different lengths [5,6,15,16,19,22,50,51]. However, relying solely on morphology and single-gene methods for species identification can lead to subjective biases and ambiguous or even incorrect delineations, especially when the number of samples is small and the sequence qualities are low [52,53]. This is particularly common in truffle species. For example, Asian black truffles such as *T. indicum*, *T. himalayense*, *T. sinense* and *T. formosanum* are generally poorly defined due to morphological similarities and variable sequence quality [33,54]. Although some studies speculate that *T. indicum* is *T. sinense*, this conclusion is not yet supported by molecular evidence from type specimens [6]. Taking into account this scenario, there is an urgent need to carry out an integrative approach combining morphological and molecular information in order to have deeper insights into the Melanosporum group. Therefore, we collected a series of samples from truffles belonging to this latter group and conducted initial classification based on morphological features. Subsequently, we conducted molecular analysis and sequenced four different regions for phylogenetic analysis. As a consequence, in this work, we describe three new species and redefined some other species, validating their taxonomic status. The results indicated that in some cases, the molecular and morphological characterization coincide, further supporting initial classifications. For example, species with depressions belonged to the so-called subgroups 1, 3, and 4, while species without depressions were all clustered in another subgroup called 2. However, in other cases, inconsistencies between morphological and molecular data were observed, suggesting that further insights are needed related to species diversity assessment (e.g., *T. pseudobrumale* and *T. melanoexcavatum*). Our study contributes to the knowledge of the taxonomic status of some species by adding the examination of more specimens and their morphological and molecular characterization. 

Currently, there are at least 14 species in the Melanosporum group. Our phylogenetic tree based on a concatenated multilocus dataset revealed that the species belonging to this group are nested in four phylogenetic clades or subgroups. Subgroup 1 includes six species, *T. pseudobrumale*, *T. melanoexcavatum*, *T. microexcavatum* sp. nov., *T. pseudohimalayense*, *T. variabilisporum*, and *Tuber* sp. 5. *Tuber microexcavatum* sp. nov. was molecularly identified as a new species on the basis of less than 80% ITS similarity to any known species. Although *T. microexcavatum* has lower maturity spores, it can also be distinguished from the closely related species *T. pseudobrumale* by ascospore size, surface warts, and spore number.

We also re-evaluated and delimited the phylogenetic relationship between *T. pseudobrumale* and *T. melanoexcavatum* in subgroup 1. Molecularly, the types of *T. pseudobrumale* and *T. melanoexcavatum* and 22 examined specimens in this work, are highly clustered into a single branch, and the ITS similarities of both species sequences were greater than 97.9%.

Based on molecular data analysis, *T. pseudobrumale* and *T. melanoexcavatum* are closely related phylogenetically. ITS rDNA sequence analysis showed that these species have similarities ranging from 98.0% to 99.7%. Morphologically, these two species share common features in the original descriptions, such as the ellipsoidal shape of the spores. The only difference is the number of spores within the asci. *Tuber pseudobrumale* has ellipsoidal spores (Q = 1.27) and 3–7 spored asci according to Li et al. [42], while *T. melanoexcavatum* has 5–8 spored asci, and its spores are also ellipsoidal (Q = 1.19) [6]. It is noteworthy that *T. pseudobrumale* is assigned only one Q value (1.27) in the aforementioned article, whereas *T. melanoexcavatum* has also only one Q value (1.19). This later Q value should correspond to subglobose spores, however, in their original description, Wang et al. (2020) reported spore sizes as follows “… ascospores in 5-spored asci 22–24.7 × 15.4–16.9 μm, 6-spored asci 21.4–24 × 14.3–16.0 μm, 7-spored asci 20–22 × 13.5–15.5 μm, 8-spored asci 18.7–21.2 × 12.6–15.1 μm …”, which indicates that the spores were ellipsoid rather than subglobose. In order to elucidate whether *T. pseudobrumale* and *T. melanoexcavatum* may be different or the same species, we incorporated our own collected specimens with a detailed morphological characterization and analyzed previously published information. As a result, we were able to understand the morphological characteristics of *T. pseudobrumale* more comprehensively (Figure 5), including spore shape and Q value, which could adequately cover the aforementioned features. Based on these morphological, molecular, and phylogenetic analyses, it is possible to conclude that *T. melanoexcavatum* is a synonym of *T. pseudobrumale*.

Another two new species, *T. yunnanense* and *T. melanoumbilicatum*, were identified within subgroups 2 and 4, respectively. Phylogenetically, *T. yunnanense* belongs to subgroup 2, a clade that includes *T. yigongense*, *T. sinense*, *T. formosanum*, *T. longispinosum*, *T. melanosporum,* and *T. regimontanum*; they have pyramidal warts and no cavities in their ascomata. The ITS similarities between *T. yunnanense* and species of subgroup 2 were 87.0–93.0%. Morphologically, *T. yunnanense* is similar to *T. longispinosum* in the number of spores per asci having 1–5(–6) spiny spores, with a few asci possessing short stalks. However, they differ in spore color and size. The color of *T. yunnanense* is golden, whereas that of *T. longispinosum* is brown to dark brown. *T. yunnanense* spores are (20–)22–44(–55) × (12–)14–29(–35) μm, Q = 1.1–2.2, and those of *T. longispinosum* are (15–)21–35(–41) × (12–)15–26(–30) μm, Q = 1.0–2.1 [32]. The spores of *T. yunnanense* are mainly long shuttle-shaped (Qm = 1.74 ± 0.1), occasionally subglobose, whereas those of *T. longispinosum* are mainly ellipsoid, occasionally globose, and long shuttle-shaped. Subgroups 3 and 4 include *T. brumale* and *T. melanoumbilicatum*, respectively, both of which had concave ascomata. Molecular analysis reveals that the ITS similarity between these two species is 70%. In morphology, *T. melanoumbilicatum* resembles *T. pseudobrumale* in having a distinct basal cavity on the surface of ascomata and asci with multiple spores [41]. However, they differ significantly in spore shape. The spores of *T. melanoumbilicatum* are long oval with Qm = 1.65 ± 0.1, whereas those of *T. pseudobrumale* are ellipsoidal with Qm =1.43 ± 0.2.

The presence of 14 phylogenetic species in the Melanosporum group was confirmed by the phylogenetic tree based on the ITS, LSU, *tef1*-α, and *rpb2* dataset (Figure 1). The Melanosporum group currently comprises 16 species, with 14 of them well supported by reliable molecular data, 13 of which are well-defined, and the status of the other three needs to be clarified by studying more collections, conducting more detailed morphological and molecular analyses. Among these, the species in subgroups 1, 3 and 4 all have distinct concavities. In the genus *Tuber*, the Melanosporum and Excavatum group have distinct cavities on the surface of the ascomata. This special surface structure may reflect the specialized adaptations of the Melanosporum and Excavatum groups in relation to their life histories and ecological environments [55]. Species of the Melanosporum and Excavatum groups, which typically grow in soil, have adapted to different habitat conditions. This adaptation likely involves the regulation of gas exchange, water uptake, and nutrient acquisition through their cavity structures [16]. Such morphological features may have gradually evolved, providing these fungi with effective mechanisms for survival and reproduction in environments with different conditions. Further studies on these cavity structures could reveal the biological characteristics and adaptive mechanisms of species of the Melanosporum and Excavatum groups. This research thus provides new insights and perspectives for the study of fungal taxonomy and ecology of the Melanosporum group of truffle species.

Key to the taxa of Melanosporum group
1. Ascomata has a cavity21. Ascomata no a cavity32. Ascomata yellowish brown***T. microexcavatum***2. Ascomata black43. Spore shape, Q > 1.753. Spore shape, Q < 1.774. Spore shape, broadly ellipsoidal or ellipsoidal64. Spore shape, long ellipsoid85. Spore shape, mainly long shuttle-shaped***T. yunnanense***5. Spore shape, mainly ellipsoid96. Spore shape, broadly ellipsoidal*T. pseudohimalayense*6. Spore shape, mainly ellipsoid107. Height of spore ornamentation, 3–6(–7) µm, spiny*T. sinense*7. Height of spore ornamentation, 2–5(–6) µm, spino-reticulate*T. formosanum*8. Height of spore ornamentation, 3.4 ± 0.38 µm, spino-reticulate*T. brumale*8. Height of spore ornamentation, 0.9–8.2 µm, spino-reticulate***T. melanoumbilicatum***9. Height of spore ornamentation, spiny*T. longispinosum*9. Height of spore ornamentation, spino-reticulate1110. Height of spore ornamentation, 0.4–9.6 µm, spino-reticulate*T. pseudobrumale*10. Height of spore ornamentation, spino-reticulate1211. Height of spore ornamentation, 2.5–4 µm, densely spino-reticulate*T. yigongense*11. Height of spore ornamentation, spino-reticulate1312. Spore length and width, 15–20 × 10–15 μm*Tuber* sp. 512. Spore length and width, 16.5–37.5 × 13.25–27.5 μm*T. variabilisporum*13. Spore length and width, 28–55 × 18–31 μm*T. regimontanum*13. Spore length and width, 28–32 × 16–21 μm*T. melanosporum*

## Data Availability

A publicly available dataset was analyzed in this study. The resulting alignments were deposited in TreeBASE (http://www.treebase.org; accession number 31641 (accessed on 22 August 2024))). All newly generated sequences were deposited in GenBank (https://www.ncbi.nlm.nih.gov/genbank/ (accessed on 12 October 2023), mentioned in the text in Table 1 and Table A1 and in Figure 1). All new taxa were deposited in MycoBank (https://www.mycobank.org/ (accessed on 21 May 2024)).

## Appendix A

**Table A1:** Species, lineage voucher specimen, country, and corresponding GenBank accessions of the *Tuber* specimens used for the phylogenetic analysis.

Taxon Name	Lineage	Voucher Specimen	Country	ITS	LSU	*tef1*-α	*rpb2*
*Choiromyces sichuanensis*	outgroup	YNAU003, holotype	Sichuan, China	MW380902	OK576632	PP092037	OR832439
*Choiromyces sichuanensis*	outgroup	YNAU004	Sichuan, China	OK585070	OK576633	PP092038	OR832440
*Tuber aestivum*	aestivum	GB202	Italy	–	JQ925679	JX022565	JQ954487
*Tuber aestivum*	aestivum	JT30500	Sweden	HM485340	–	–	JQ954488
*Tuber borchii*	puberulum	BJTC FAN217	New Zealand	KT067681	KT067706	KT067717	OM584229
*Tuber borchii*	puberulum	GB1/GB32	Italy	FJ809852	FJ809799	JX022571	JQ954492
*Tuber brumale*	melanosporum	GB52	Italy	HM485345	JQ925683	–	JQ954494
*Tuber brumale*	melanosporum	GB53	Italy	FJ748900	JQ925684	–	JQ954495
*Tuber californicum*	puberulum	JT28058	USA	HM485346	JQ925685	JX022574	JQ954496
*Tuber canaliculatum*	macrosporum	JT28215	USA	JQ925643	–	JX022575	JQ954497
*Tuber canaliculatum*	macrosporum	OSC59072	USA	HM485347	–	JX022576	JQ954498
*Tuber crassitunicatum*	rufum	BJTC FAN465, holotype	Yunnan, China	MH115295	OM366205	OM649610	OM584268
*Tuber depressum*	excavatum	BJTC FAN340	Sichuan, China	OM256744	OM366187	OM649592	OM584250
*Tuber depressum*	excavatum	BJTC FAN534	Sichuan, China	OM256764	OM366211	OM649616	–
*Tuber dryophilum*	puberulum	GB35	Italy	JQ925644	JQ925687	JX022577	–
*Tuber dryophilum*	puberulum	GB37	Italy	HM485354	JQ925688	JX022578	JQ954501
*Tuber formosanum*	melanosporum	BJTC FAN107	Yunnan, China	MF621549	OM366159	OM649564	OM584210
*Tuber formosanum*	melanosporum	BJTC FAN356	Sichuan, China	MF627986	OM366189	OM649594	OM584252
*Tuber glabrum*	macrosporum	BJTC FAN228, holotype	Yunnan, China	KF002731	OM366177	OM649581	OM584234
*Tuber glabrum*	macrosporum	BJTC FAN232, paratype	Yunnan, China	KF002727	OM366179	OM649583	OM584236
*Tuber huidongense*	rufum	BJTC FAN104	Yunnan, China	JF921163	OM366158	OM649563	OM584209
*Tuber huidongense*	rufum	BJTC FAN101	Yunnan, China	OM311172	OM366156	OM649562	OM584208
*Tuber jinshajiangense*	puberulum	BJTC FAN406	Yunnan, China	KX575841	OM366199	OM649604	OM584262
*Tuber jinshajiangense*	puberulum	BJTC FAN407	Yunnan, China	KX575842	OM366200	OM649605	OM584263
*Tuber liaotongense*	rufum	BJTC FAN550	Beijing, China	MH115302	OM366213	OM649618	OM584272
*Tuber lijiangense*	puberulum	BJTC FAN307	Yunnan, China	KP276188	KP276203	KP276206	OM584244
*Tuber lishanense*	rufum	BJTC FAN718, holotype	Shanxi, China	MH115303	MH115304	OM649622	OM584276
*Tuber lishanense*	rufum	BJTCFAN683	Shanxi, China	MH115305	MH115306	OM649621	OM584275
*Tuber longispinosum*	melanosporum	K225	Japan	AB553414	AB553518	AB553538	AB553558
*Tuber longispinosum*	melanosporum	K447, holotype	Japan	AB553423	–	–	–
*Tuber macrosporum*	macrosporum	JT13362	Italy	HM485373	FJ809838	JX022590	–
*Tuber magnatum*	aestivum	GB12	Italy	JQ925645	JQ925700	JX022591	JQ954512
*Tuber magnatum*	aestivum	GB13	Italy	JQ925646	JQ925701	JX022592	JQ954513
*Tuber melanoexcavatum*	melanosporum	YAAS L3605, holotype	Yunnan, China	KY081684	–	–	–
*Tuber melanoexcavatum*	melanosporum	YAAS L3636	Yunnan, China	KY081685	–	–	–
*Tuber melanosporum*	melanosporum	GB200	Italy	FJ748904	JQ925703	JX022594	JQ954515
*Tuber melanoumbilicatum*	melanosporum	YNAU017, holotype	Yunnan, China	OK625304	OR661815	OR832379	OR832411
*Tuber melanoumbilicatum*	melanosporum	YNAU018	Yunnan, China	OK625305	OR661816	OR832380	OR832412
*Tuber microexcavatum*	melanosporum	YNAU1263	Yunnan, China	OR250184	OR661838	OR832381	OR832413
*Tuber microexcavatum*	melanosporum	YNAU1264	Yunnan, China	OR250185	OR661839	OR832382	OR832414
*Tuber neoexcavatum*	excavatum	BJTC FAN184, holotype	Yunnan, China	JX458715	OM366169	OM649574	–
*Tuber neoexcavatum*	excavatum	BJTC FAN316	Yunnan, China	OM256741	OM366184	OM649589	OM584247
*Tuber oligospermum*	puberulum	AH38984	Spain	JN392261	JN392320	–	–
*Tuber oligospermum*	puberulum	AH37783	Spain	JN392260	JN392324	–	–
*Tuber pseudobrumale*	melanosporum	YAAS L3181, holotype	Yunnan, China	KJ742703	–	–	–
*Tuber pseudobrumale*	melanosporum	BJTC FAN306	Yunnan, China	OM287838	OM366183	OM649587	OM584243
*Tuber pseudobrumale*	melanosporum	BJTC FAN322	Yunnan, China	OM287839	OM366186	OM649591	OM584249
*Tuber pseudobrumale*	melanosporum	YNAU0126	Yunnan, China	OR665398	OR825717	OR832383	OR832415
*Tuber pseudobrumale*	melanosporum	YNAU0127	Yunnan, China	OR665399	OR661817	OR832384	OR832416
*Tuber pseudobrumale*	melanosporum	YNAU0128	Yunnan, China	OR665400	OR661818	OR832385	OR832417
*Tuber pseudobrumale*	melanosporum	YNAU0145	Yunnan, China	OR665401	OR661819	OR832386	OR832418
*Tuber pseudobrumale*	melanosporum	YNAU0148	Yunnan, China	OR665402	OR661820	OR832387	OR832419
*Tuber pseudobrumale*	melanosporum	YNAU0149	Yunnan, China	OR665403	OR661821	OR832388	OR832420
*Tuber pseudobrumale*	melanosporum	YNAU0150	Yunnan, China	OR665404	OR661822	OR832389	OR832421
*Tuber pseudobrumale*	melanosporum	YNAU0151	Yunnan, China	OR665405	OR661823	OR832390	OR832422
*Tuber pseudobrumale*	melanosporum	YNAU0152	Yunnan, China	OR665406	OR661824	OR832391	OR832423
*Tuber pseudobrumale*	melanosporum	YNAU0179	Yunnan, China	OR665407	OR661825	OR832392	OR832424
*Tuber pseudobrumale*	melanosporum	YNAU0180	Yunnan, China	OR665408	OR661826	OR832393	OR832425
*Tuber pseudobrumale*	melanosporum	YNAU0181	Yunnan, China	OR665409	OR661827	OR832394	OR832426
*Tuber pseudobrumale*	melanosporum	YNAU0221	Yunnan, China	OR665410	OR661828	OR832395	OR832427
*Tuber pseudobrumale*	melanosporum	YNAU0228	Yunnan, China	OR665411	OR661829	OR832396	OR832428
*Tuber pseudobrumale*	melanosporum	YNAU0229	Yunnan, China	OR665412	OR661830	OR832397	OR832429
*Tuber pseudobrumale*	melanosporum	YNAU0230	Yunnan, China	OR665413	OR661831	OR832398	OR832430
*Tuber pseudobrumale*	melanosporum	YNAU0233	Yunnan, China	OR665414	OR661832	OR832399	OR832431
*Tuber pseudobrumale*	melanosporum	YNAU0240	Yunnan, China	OR665415	OR661833	OR832400	OR832432
*Tuber pseudobrumale*	melanosporum	YNAU0275	Yunnan, China	OR665416	OR661834	OR832401	OR832433
*Tuber pseudobrumale*	melanosporum	YNAU0276	Yunnan, China	OR665417	OR661835	OR832402	OR832434
*Tuber pseudobrumale*	melanosporum	YNAU0277	Yunnan, China	OR665418	OR661836	OR832403	OR832435
*Tuber pseudobrumale*	melanosporum	YNAU0885	Yunnan, China	OR665419	OR661837	OR832404	OR832436
*Tuber pseudofulgens*	excavatum	BJTC FAN399, holotype	Yunnan, China	OM256757	OM366196	OM649601	OM584259
*Tuber pseudofulgens*	excavatum	BJTCFAN388	Sichuan, China	OM256755	OM366194	OM649599	OM584257
*Tuber pseudohimalayense*	melanosporum	YNAU1608	Yunnan, China	PP784763	PP784589	PP796851	PP796861
*Tuber pseudohimalayense*	melanosporum	BJTC FAN122	Sichan, China	MF627983	OM366162	OM649567	OM584213
*Tuber pseudohimalayense*	melanosporum	YNAU1663	Yunnan, China	PP784764	PP784590	PP796852	PP796862
*Tuber pseudohimalayense*	melanosporum	YNAU1664	Yunnan, China	PP784765	PP784591	PP796853	PP796863
*Tuber pseudohimalayense*	melanosporum	YNAU1665	Yunnan, China	PP784766	PP784592	PP796854	PP796864
*Tuber pseudohimalayense*	melanosporum	YNAU1666	Yunnan, China	PP784767	PP784593	PP796855	PP796865
*Tuber pseudohimalayense*	melanosporum	YNAU1344	Yunnan, China	PP784768	PP784594	PP796856	PP796866
*Tuber puberulum*	puberulum	ZB1433	Hungary	JF261382	JF261346	–	–
*Tuber puberulum*	puberulum	ZB1983	Hungary	JF261390	JF261357	–	–
*Tuber regimontanum*	melanosporum	ITCV 909	Mexico	EU375838	FJ809823	JX022600	JQ954520
*Tuber shidianense*	puberulum	HKAS 88770, holotype	Yunnan, China	KT444595	KY174960	OR832405	OR832437
*Tuber shidianense*	puberulum	HKAS 88771, paratype	Yunnan, China	KT444596	KY174961	OR832406	OR832438
*Tuber sinense*	melanosporum	BJTC FAN108	China	MF627968	OM366160	OM649565	OM584211
*Tuber sinense*	melanosporum	BJTC FAN110	China	MF627970	OM366161	OM649566	OM584212
*Tuber sinoaestivum*	aestivum	BJTC FAN522	Sichuan, China	OM256774	OM366210	OM649615	OM584271
*Tuber sinoaestivum*	aestivum	BJTC FAN487	Yunnan, China	OM256773	OM366209	OM649614	OM584270
*Tuber sinoexcavatum*	excavatum	BJTC FAN130, holotype	Yunnan, China	JX458717	OM366163	OM649568	OM584216
*Tuber sinoexcavatum*	excavatum	BJTC FAN166	Yunnan, China	JX458718	OM366165	OM649571	OM584221
*Tuber* sp. 1	macrosporum	K201	Japan	AB553344	AB553512	AB553532	AB553552
*Tuber* sp. 5 KA-2010	melanosporum	K229	Japan	AB553381	–	AB553536	AB553556
*Tuber* sp. 5 KA-2010	melanosporum	K403	Japan	AB553382	–	–	–
*Tuber sphaerosporum*	puberulum	JT12487	USA	FJ809853	FJ809853	JX022609	–
*Tuber taiyuanense*	rufum	BJTC FAN164	Yunnan, China	OM311182	OM366164	OM649570	OM584220
*Tuber taiyuanense*	rufum	BJTC FAN220	Yunnan, China	MH115315	OM366174	OM649578	OM584231
*Tuber umbilicatum*	rufum	BJTC FAN212	Yunnan, China	OM311201	OM366173	OM649577	OM584228
*Tuber umbilicatum*	rufum	BJTC FAN230	Yunnan, China	OM311205	OM366178	OM649582	OM584235
*Tuber variabilisporum*	melanosporum	BJTC FAN362, holotype	Sichuan, China	OM287845	OM366190	OM649595	OM584253
*Tuber variabilisporum*	melanosporum	YNAU0146	Yunnan, China	PP784759	PP784585	PP796847	PP796857
*Tuber variabilisporum*	melanosporum	YNAU0468	Yunnan, China	PP784760	PP784586	PP796848	PP796858
*Tuber variabilisporum*	melanosporum	YNAU0925	Yunnan, China	PP784761	PP784587	PP796849	PP796859
*Tuber variabilisporum*	melanosporum	YNAU1670	Yunnan, China	PP784762	PP784588	PP796850	PP796860
*Tuber wenchuanense*	rufum	BJTC FAN833	Shanxi, China	OM311256	OM366222	OM649629	OM584280
*Tuber yigongense*	melanosporum	BJTC FAN729	Tibet, China	MF663716	–	OM649623	OM584277
*Tuber yigongense*	melanosporum	BJTC FAN731, holotype	Tibet, China	MF663714	OM366216	–	–
*Tuber yunnanense*	melanosporum	YNAU019, holotype	Yunnan, China	OK625306	OR661811	OR813081	OR832407
*Tuber yunnanense*	melanosporum	YNAU020	Yunnan, China	OK625307	OR661812	OR813082	OR832408
*Tuber yunnanense*	melanosporum	YNAU0107	Yunnan, China	OR665397	OR661813	OR813083	OR832409
*Tuber yunnanense*	melanosporum	YNAU0491	Sichuan, China	OR250186	OR661814	OR813084	OR832410
